# The apical root canal system microbial communities determined by next-generation sequencing

**DOI:** 10.1038/s41598-020-67828-3

**Published:** 2020-07-02

**Authors:** Luciana Carla Neves de Brito, Janet Doolittle-Hall, Chun-Teh Lee, Kevin Moss, Wilson Bambirra Júnior, Warley Luciano Fonseca Tavares, Antônio Paulino Ribeiro Sobrinho, Flávia Rocha Fonseca Teles

**Affiliations:** 1 School of Dentistry, University of Itauna, Itaúna, MG Brazil; 2 Dental Research/Center for Oral Systemic Diseases, School of Dentistry, University of North Carolina at Chapel Hill, Chapel Hill, NC USA; 3 Department of Periodontics and Dental Hygiene, School of Dentistry, The University of Texas Health Science Center at Houston, Houston, TX USA; 4 Department of Operative Dentistry, School of Dentistry, Federal University of Minas Gerais, Belo Horizonte, MG Brazil; 5 School of Dental Medicine, School of Engineering and Applied Sciences, Center for Innovation and Precision Dentistry, University of Pennsylvania, Philadelphia, USA

**Keywords:** Chemical biology, Microbiology, Pathogenesis

## Abstract

The aim of this study was to explore the microbial communities of endodontic infections at their apical portion by 16S rRNA Illumina sequencing and delineate the core microbiome of root canal infections and that of their associated clinical symptomatology. Samples were collected from fifteen subjects presenting one tooth with a root canal infection, and their associated symptoms were recorded. Samples were collected from the apical third of roots using a #10 K file and then amplified using multiple displacement amplification and PCR-amplified with universal primers. Amplicons were sequenced (V3–V4 hypervariable region of the 16S rRNA gene) using MiSeq (Illumina, CA). The microbial composition of the samples was determined using QIIME and HOMINGS. Data were analyzed using *t* tests and ANOVA. A total of 1,038,656 good quality sequences were obtained, and OTUs were assigned to 10 bacterial phyla, led by *Bacteroidetes* (51.2%) and *Firmicutes* (27.1%), and 94 genera were represented primarily by *Prevotella* (17.9%) and *Bacteroidaceae* G-1 (14.3%). Symptomatic teeth were associated with higher levels of *Porphyromonas* (*p* < 0.05) and *Prevotella*. *P. endodontalis* and *P. oris* were present in both cores. The present study demonstrated the complexity of the root canal microbiome and the “common denominators” of root canal infections and identified taxa whose virulence properties should be further explored. The polymicrobial etiology of endodontic infections has long been established. However, few studies have focused on expanding the breadth and depth of coverage of microbiome-infected root canals at their apical portion.

## Introduction

Microbiological evaluations of infected root canals have expanded our knowledge on the topic^1–4^, confirmed the predominance of anaerobic species, revealed previously unrecognized bacterial diversity^1–3,5,6^ and showed that the complexity of the microbial consortium influences the pathogenesis of periradicular conditions^4,7^. In infected root canals, microbial communities remain as surface-associated biofilms^8,9^. The bacterial biofilm requires treating root canals to prevent and/or heal apical periodontitis^10^.

There is overwhelming evidence to support that an unspecific microbial community is able to induce periapical lesion development^4,7,11^. However, as stated by Tatikonda et al.^12^, there are a few studies that focus on analyzing “the pulp canal segments”. The infected apical third of root canals maintains a distinct array of microorganisms from its coronal segment^13,14^. Nevertheless, few studies have focused on the analysis of the most apical portion of endodontic infections. This gap in the current literature has precluded a better picture of the root canal core microbiome and its associated clinical parameters.

The cloistered root canal space interferes in its microbial colonization (10, 12). The microbial status of infected root canals was demonstrated by studies that have progressed depending on the evolution of microbiological methods. Recently, culture-independent strategies, such as next-generation sequencing, have improved this knowledge. Additionally, molecular studies have revealed significant differences in the prevalence of certain pathogens, demonstrating that geographical and individual characteristics may influence bacterial community profiles^1,2,5,15–18^. Next-generation sequencing (NGS) has revolutionized genomic research^19,20^. The ultramodern MiSeq platform is ideal for rapid and cost-effective genetic analysis^21^. However, MiSeq sequencing may not always reach species-level taxonomic resolution. To improve this limitation, HOMINGS^22–24^ utilized the speed and efficiency of next-generation sequencing combined with the refinement of bacterial species-level identification based on 16S rDNA comparisons^17^.

Therefore, knowing that the apical portion of the root canal infection harbors a distinct microbiome from its coronal segment, this study aimed to explore the microbiome of the apical portion of the root canal through metagenetics approaches and its association with clinical symptomatology.

## Materials and methods

### Study population

Study participants were 15 patients referred to the dental school to receive endodontic care. The exclusion criteria for this study was antibiotic therapy up to 3 months before starting endodontic therapy, systemic diseases, and pregnancy. All participants signed the Free Agreement Formulary. The Ethics Committee of the FUMG approved this study (ETIC 122⁄08). Clinical samples were taken from 15 teeth (single and multirooted) with pulp necrosis and apical periodontitis that were diagnosed by clinical (presence of tissue swelling, percussion sensitivity, symptomatic or asymptomatic) and radiographic analyses, in addition to pulp sensibility tests.

Additionally, if the tooth was symptomatic, the following parameters were recorded: onset of pain (time and duration) and quality of pain (throbbing, stabbing, dull) and whether teeth were single or multirooted. The final diagnosis was made based on those findings. All teeth selected for sampling presented pulpal necrosis, as well as no history of trauma, periodontal involvement, or previous root canal treatment. The clinical parameter of each tooth is described in Supplemental Table 1.

### Root canal samples

The selection and preparation of the teeth, as well as the sample collection, was performed, as previously described^1,2^, by the same experienced endodontist. Briefly, the tooth was cleaned with pumice and isolated with a rubber dam. The teeth were decontaminated and disinfected with a 30% hydrogen peroxide solution (H_2_O_2_) and then with 2.5% sodium hypochlorite solution (NaOCl). The access cavity was prepared with a high speed sterile carbide bur, and before the pulp chamber was exposed, the cleaning of the tooth and rubber dam was repeated as previously described. The samples were taken by filing the root canal walls with a sterile #10 K-type hand file (Maillefer, Ballaigues, Switzerland). The file was introduced into the canal to the level of the tooth apex. The tooth length was defined using an apex locator (Root ZXII ®; J. Morita-USA, Irvine, CA, USA). In multirooted teeth, samples were collected from the largest root canal. After removal from the canal, the final 4 mm of the file was cut using a sterile pair of surgical scissors and placed in a microcentrifuge tube containing 20 μl of alkaline lysis buffer (400 mM KOH, 100 mM dithiothreitol, 10 mM EDTA). After 10 min of incubation on ice, 20 μl of neutralization solution (400 mM HCl, 600 mM Tris–HCl, pH 0.6) was added. Samples were kept at 4 °C until analysis.

### Multiple displacement amplification (MDA) of root canal samples

To ensure the availability of adequate DNA for analysis, Multiple displacement amplification (MDA) was performed prior to sequencing, as previously described^1,2,25^. The Illustra GenomiPhi V2 DNA Amplification Kit (GE Healthcare, Salt Lake City, UT, USA) was used, and DNA measurement prior to and after amplification was performed using the Picogreen™ dsDNA quantification assay (Invitrogen, Carlsbad, CA, USA). An example of the similarity of samples before and after MDA can be observed in Supplemental Figure 1.

### Illumina sequencing of barcoded 16S rRNA gene amplicons

Sample DNA was analyzed by sequencing the 16S rRNA gene V3–V4 hypervariable region using MiSeq (Illumina, CA), according to the protocol described by Caporaso et al.^26^. In brief, 10–50 ng of DNA was PCR-amplified using the 341F/806R universal primers targeting the V3–V4 hypervariable region: 341F (forward) AATGATACGGCGACCACCGAGATCTACACTATGGTAATTGTCCTACGGGAGGCAGCAG; 806R (reverse) CAAGCAGAAGACGGCATACGAGAT**TCCCTTGTCTCC **AGTCAGTCAGCCGGACTACHVGGGTWTCTAAT, where the ‘TCCCTTGTCTCC’ region represents the appropriate barcode sequences and the underlined bases make the PCR products Illumina sequencing compatible^22–24^. PCR samples were purified using AMPure beads, and 100 ng of each barcoded library was pooled, purified and quantified using a bioanalyzer and qPCR. Then, 12 pM of each library mixture library spiked with 20% PhiX was loaded onto the MiSeq and sequenced.

### Sequencing analytical pipeline

The reads generated using MiSeq were analyzed using the QIIME pipeline^27^. In brief, the quality control of the reads was performed using FastQC. The paired-end reads were merged using Flash. The libraries were split in QIIME according to the barcodes used in the sequencing run, low-quality reads were filtered out, and chimeras were removed using UCHIME. Operational taxonomic units (OTUs) were picked using the Human Oral Microbiome Database (HOMD) v13.2 as a reference database^28^ using a 97% similarity threshold. Taxonomy was assigned using the Ribosomal Database Project (RDP) classifier trained on the HOMD v13.2 database with assignments required to meet a > 80% confidence threshold.

Because MiSeq sequencing may not always reach species-level taxonomic resolution, in the present study, we complemented it with HOMINGS^22–24^ an in silico 16S rDNA probe analysis that allows for species-level identification of sequencing datasets generated with MiSeq (https://homings.forsyth.org)^17^. Species-specific, 16S rRNA-based oligonucleotide “probes” were used in a Perl program based on a text string search to identify the frequency of oral bacterial targets. HOMINGS comprises 671 oligonucleotide probes of 17–40 bases that target 538 individual oral bacterial species/phylotypes or, in some cases, a few closely related taxa^22–24^.

### Data analysis

Taxa detected by HOMINGS were mapped to species- and genus-level targets (v2.0, https://homings.forsyth.org/bacterialtaxa.html). Analyses were performed at the species level or by summing the relative abundance of the taxa detected to the genus or phylum level.

The HOMINGS taxa detected at ≥ 0.1% relative abundance in ≥ 50% of all samples were taken to constitute the microbial communities, which were subdivided based on the mean relative abundance of the taxa in symptomatic and asymptomatic samples^29^. Taxa that were present in ≥ 50% of samples in any of the clinical categories but were not part of the microbial communities considering all samples constituted microbial communities of those conditions. These taxa were subgrouped based on the mean relative abundance of the taxa in symptomatic and asymptomatic samples. Data analyses were performed using R 3.2.1 (https://cran.r-project.org/). The microbial composition of the symptomatic and asymptomatic samples was compared using *t *tests and ANOVA. Due to the exploratory nature of the study, no adjustments for multiple comparisons were performed.

## Results

Study participants had a mean age of 39.8 years old (SD = 13.8; range: 12–69 years old), and most of them were female (n = 10). Most of the sampled teeth were multirooted (n = 12) and were symptomatic (n = 9) or presented with a closed cavity (n = 11).

**Figure 1:**
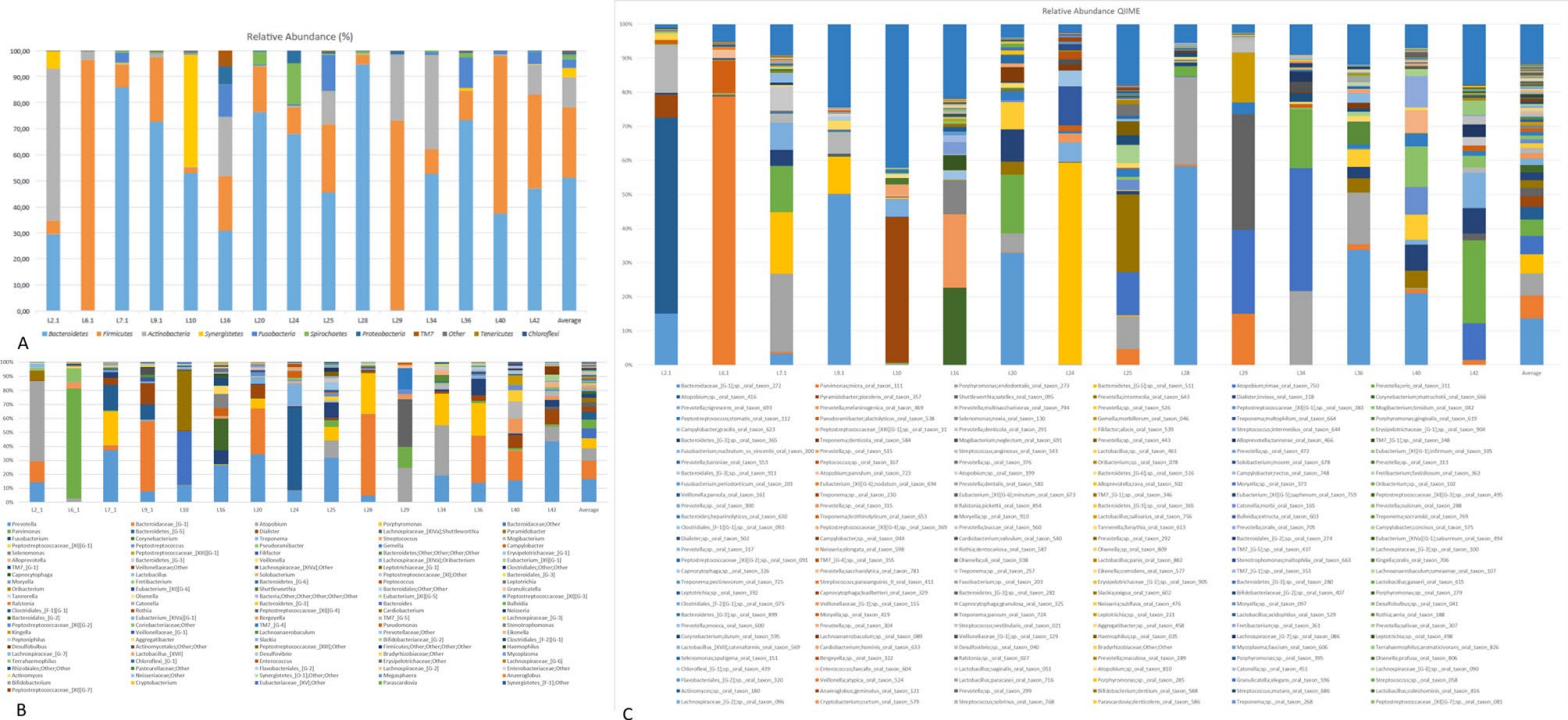
Bar charts of the composition of each of the samples examined and their average representation at the phylum (**A**), genus (**B**) and species levels (**C**). Species-level results were obtained using HOMINGS. The percentage of reads that were not identified by HOMINGS (i.e., unassigned reads) was not plotted.

A total of 1,038,656 sequences were obtained from the 15 samples after quality control (median: 29,328 reads). A total of 946 OTUs were identified and assigned to 10 phyla, 94 genera and 311 species using the QIIME pipeline. The most abundant phylum detected was *Bacteroidetes* (51.2%), followed by *Firmicutes* (27.1%) and *Actinobacteria* (11.5%) (Fig. 1a). The most prominent genera were *Prevotella* (17.9%) and *Bacteroidaceae* G-1 (14.3%) (Fig. 1b), while *Bacteroidaceae* [G-1] sp oral taxon (ot) 272 (12.6%), *Parvimonas micra* (6.2%), *Porphyromonas endodontalis* (3.4%), and *Bacteroidetes* [G-5] sp ot 511 (2.5%) were the most numerous species/phylotypes (Fig. 1c). Despite being a close-ended analysis, HOMI*NGS* detected the most representative taxa in the samples, covering, on average, 84.1% of the post QC MiSeq reads (range: 57–93%).

**Figure 2:**
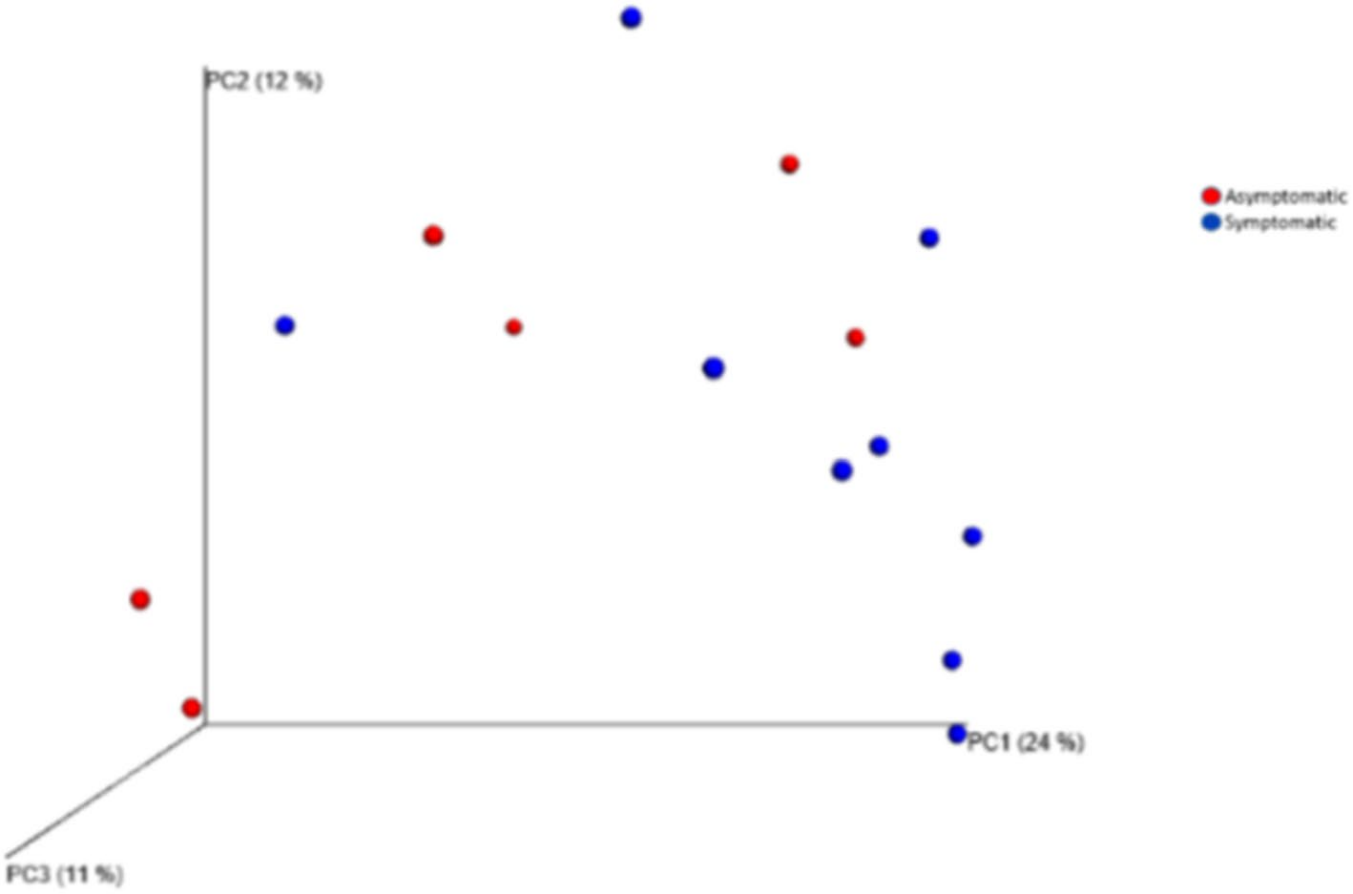
Principal coordinate analysis (PCoA) based on unweighted UniFrac distances obtained from the QIIME analytical pipeline for cavity status (a) and the presence of symptoms (b).

**Figure 3:**
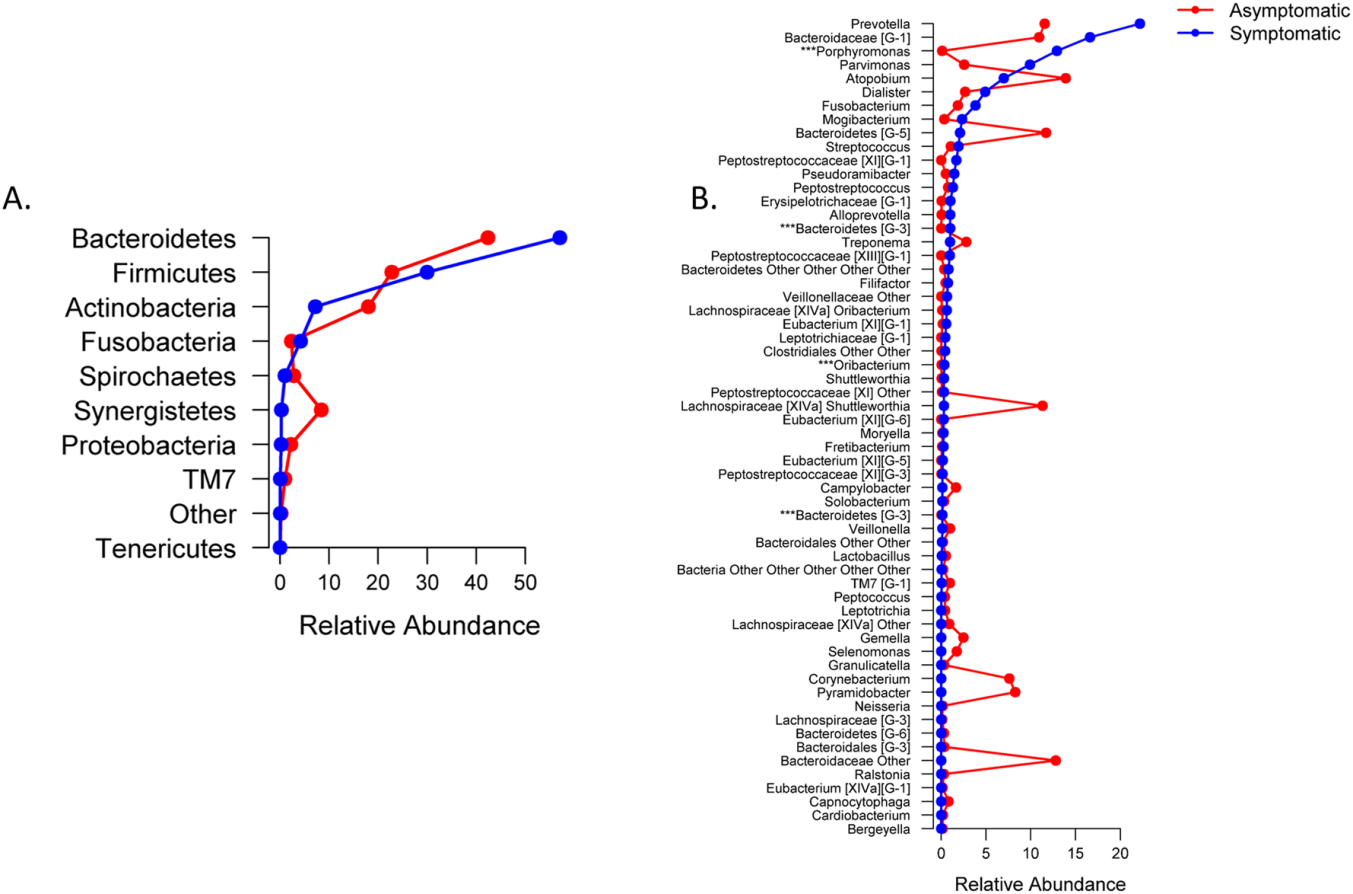
Line plots of the microbial composition of the samples in which symptoms were present (blue) or absent (pink), at the phylum (**A**), genus (**B**) and species levels (**C**). Graphs show the mean relative abundance for phyla that were 0.01% different, genera that were 0.1% different, and species that were 0.2% different. Taxa were sorted according to relative abundance in the positive group. ***Taxa with statistically significant differences (*p* ≤ 0.05).

Principal coordinate analysis (PCoA) suggested that the status of the cavity at the time of sampling (open/closed cavity) not only can influence (or be influenced by) the microbial composition of the local biofilm, but this effect is of a lesser magnitude regarding the presence of symptoms because limited clustering was observed in those cases (Fig. 2a, b). The phyla *Bacteroidetes* and *Firmicutes* predominated in symptomatic and asymptomatic cases (Fig. 3a). In the presence of symptoms, higher levels of *Prevotella* (absence × presence, 11.5% × 22.2%), *Bacteroidaceae* [G-1] (10.9% × 16.6%), *Porphyromonas* (0.1% × 12.9%; *p* < 0.05), *Parvimonas* (2.6% × 9.9%) and *Dialister* (2.7% × 4.9%) were observed (Fig. 3b). *Bacteroidaceae *[G-1] sp ot 272 (9.9% × 14.4%), *P. micra* (2.4% × 8.8%), *Prevotella oris* (0.1% × 7.9%, *p* < 0.05), *P. endodontalis* (0.0% × 5.6%), *Porphyromonas* sp ot 395 (0.0% × 4.9%) and *D. invisus* (0.2% × 3.6%; *p* < 0.05) were the predominant species/phylotypes (Fig. 3c).

The presence of an open cavity at the time of sampling (Supplemental Figure 2a, b, c) was associated with higher levels of *Bacteroidetes* (*p* < 0.05), whereas teeth that were closed harbored more *Actinobacteria* (*p* < 0.05). Furthermore, open teeth presented a higher abundance of *Bacteroidaceae* [G-1] sp (closed × open; 8.6% × 30.1%), *Prevotella* sp (16.0% × 23.2%), *Porphyromonas* sp (5.7% × 13.7%), *Dialister* (2.3% × 8.6%, *p* < 0.05) and *Filifactor* (0.3% × 1.5%, *p* < 0.05). In particular, those types of teeth had higher levels of *Bacteroidaceae* G1 ot 272 (7.6% × 26.3%), *P. endodontalis* (0.9% × 10.2%), *P. oris* (3.6% × 8.0%), *D. invisus* (1.5% × 4.2%) and *D. pneumosintes* (0.8% × 4.0%) (Supplemental Figure 2). Teeth with closed cavities were rich in *Atopobium* (13.2% × 0.4%, *p* < 0.05) and *Parvimonas* (9.3% × 0.6%), particularly *P. micra* (9.3% × 0.6%).

**Figure 4:**
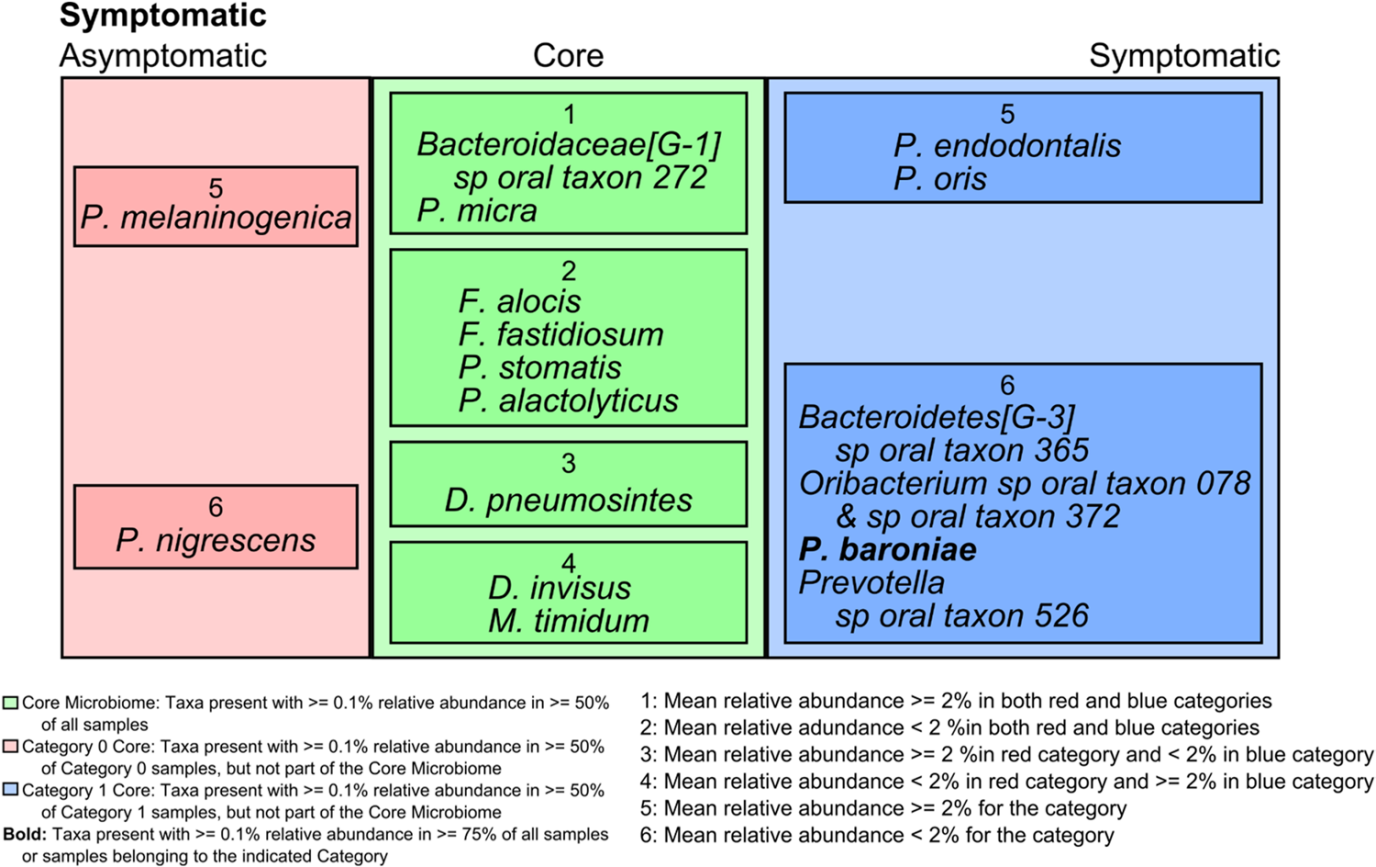
Core microbiome of the samples analyzed according to the clinical symptomatology studied. The HOMINGS probes that were present with ≥ 0.1% relative abundance in ≥ 50% of all samples constitute the core microbiome (green). Samples were divided into two categories based on the absence (0) or presence (1) of symptoms. The core microbiome was subdivided into 2 groups based on the mean relative abundance of the taxa in samples in each clinical category (presence or absence of symptomatology). Furthermore, taxa that were present in ≥ 50% of samples in a single category but were not part of the core microbiome considering all samples constitute the core microbiomes. Taxa in the category core microbiomes were subgrouped based on the mean relative abundance of the taxa in samples in each clinical category. Taxa in bold were present in ≥ 75% of all samples (core) or samples in the indicated category (category cores).

Finally, we investigated the core microbiome according to the symptomatology (Fig. 4). Overall, it was composed of *Bacteriodaceae* sp ot 272, *Filifactor alocis, Fretibacterium fastidiosum, Peptostreptococcus stomatitis, Pseudoramibacter alactolyticus, D. invisus* and *D. pneumosintes.* The presence of asymptomatic teeth at the time of sampling was associated with a more diverse core than that of symptomatic cases. *P. endodontalis* and *P. oris* were consistently associated with symptoms.

## Discussion

NGS community profiling, such as pyrosequencing (the first-generation NGS approach) and Illumina (the second-generation NGS approach), allows studies to determine the microbial composition and relative abundance of taxa^10^. Pyrosequencing is a method of DNA sequencing based on the “sequencing by synthesis” principle, while Illumina improves this method in the sequencing of homopolymeric regions. Illumina technology operates by reversible terminator chemistry, presenting lower sequencing error rates and lower cost than the former method^21,30^.

In this survey, we employed MiSeq sequencing (Illumina)^31–33^ to explore the microbiome at the apical portion of endodontic infections. We obtained 1,038,656 sequences and 946 OTUs from the 15 samples (mean: 62,895 reads; range: 14,971–166,232), far surpassing pyrosequencing studies, which yield 6,000–10,000 average reads per sample^5,34^ and 187^16^, 339^20^ and 803 OTUs^5^. Recently, employing the MiSeq platform (Illumina), Sánchez-Sanhueza et al.^35^ obtained 2,248,552 reads and 86 OTUs from the 24 root canal samples.

In this study, the phyla that prevailed were similar to those in previous reports developed in Asia, Europe and Africa^5,6,20,34,36^. Conversely, reports from individuals residing on the American continent, such as the individuals who made up this study, have found *Proteobacteria*^11,16,35,37^. These contradictory results suggest that geographical conditions are not directly related to the microbial pattern since many other factors may influence its composition^35^.

Further, we combined this comprehensive microbial analysis with HOMI*NGS* species-level resolution, which represents a significant step forward in the field because most NGS-based studies of the endodontic microbiome fail to achieve species-level identification^16,20,34,38^. Overall, the most predominant genera were *Prevotella* and *Bacteroidaceae* G-1, which is in agreement with previous studies^20,36,38^. Species-level analysis indicated that *Bacteroidaceae* [G-1] sp ot 272, *P. micra*, *P. oris*, *P. endodontalis*, and *Bacteroidetes* [G-5] sp ot 511 were the most numerous species/phylotypes. Several of those taxa have been detected in association with pulpal pathology with secondary infections or have been demonstrated to be recalcitrant to treatment^2,16^. Collectively, these findings support a pathogenic role for those organisms.

The rationale for the study of symptoms of endodontic infections stems from the fact that the local microbial insult can have relevant clinical implications. We observed that the presence of symptoms has an impact on the root canal microbiome. Similar patterns have been shown previously when acute and chronic root canal infections were compared^6^. Alternatively, they might be the result of the local environmental pressures that select distinct microorganisms in each condition. Studies have endeavored to characterize the microbiota profile of different clinical endodontic conditions, such as asymptomatic or symptomatic teeth^6,15^ and the status of the coronal cavity (open/closed cavity)^39^. However, the findings were highly variable and at times, contradictory^10^. These contradictions could be attributed to differences in HVR(s) sequenced, collection methods, and OTU picking strategies.

Similar to other authors^6,34^, we found considerable variability across samples, as they presented complex combinations of taxa. Thus, the study of the microbial communities of the clinical symptomatology analyzed should help clarify taxa that are consistently associated with root canal infections. Overall, infections mainly comprise of *Bacteriodaceae* sp oral taxon 272, *F. alocis*, *F. fastidiosum*, *P. stomatitis, P. alactolyticus, P. micra*, *D. invisus* and *D. pneumosintes*. The symptom cores were characterized by *P. oris* and *P. endodontalis*, which is in line with previous reports^6,7,40–43^. Studies have reported that *Prevotella intermedia*, *Prevotella nigrescens*, *Porphymonas gingivalis*, and *Porphymonas endodontalis* are frequently detected by the use of molecular biology techniques in teeth with necrotic pulps 1,15,43. Collectively, these results suggest a prominent pathogenic role for those organisms. For instance, *F. alocis*, *F. fastidiosum*, *P. micra* and *Dialister* species have been associated with periapical infections^15^. In addition, recent studies have proposed them as candidate pathogens in periodontal diseases^44,45^. Although the study of their pathogenic mechanisms is in its infancy, it has been shown that *F. alocis* modulates microbiome and host proteome changes^46^, inhibits complement activation^48^ and induces neutrophil degranulation and chemotaxis^48^, while *D. pneumosintes* has been isolated from local^49^ and systemic infections^50^. In our core analysis, we were also able to identify new candidate pathogens, such as *Bacteriodetes sp* ot 272, 365 and *Prevotella* sp ot 526. The potential role of those phylotypes in periapical disease supports their further characterization in cultivation studies to determine their metabolism and virulence properties.

Most studies of the endodontic microbiota have employed paper points for sample collection^5,15^. Although convenient, this approach has limitations. First, they yield samples that conceivably are not representative of the apical portion of the infection, as they are likely to absorb material from the entire extension of the root canal. Second, their DNA yield appears to favor the collection of host DNA, which is detrimental to bacterial DNA^44^. Third, as demonstrated previously^51^, they can contain contaminating organisms, and their use “as a sampling tool for microbial profiling of clinical samples by open-ended techniques such as sequencing or DGGE should be avoided”. The fact that paper points might not reach the tooth apex to collect biofilm is problematic for researcher outcomes. This is a privileged location where bacteria are poised to initiate and sustain host responses that ultimately lead to signs and symptoms; hence, a prime site for studying the pathogenesis of these infections. In addition, the apical and middle coronal microbiomes present distinct profiles^14^. However, most studies that focused on this location employed extracted teeth, which preclude the routine evaluation of the microbiome of endodontic diseases. Thus, we inserted a K file into the canal to a level of the tooth apex and used the final 4 mm to analyze the microbiome, as described previously^1,2^.

In this study, one potential limitation was MDA used to increase the sample biomass prior to sequencing, as some have demonstrated its potential to introduce bias^52^. However, such potential was never demonstrated in 16S RNA sequencing studies but in metagenomics analyses. Furthermore, several recent studies have relied on MDA to study clinical isolates of *P. gingivalis*^53^ and uncultured phylotypes^54^, generating important insights into their metabolism and pathogenicity. Our group^55^ and others^56^ have used this technique to study endodontic infections, confirming previous findings obtained without MDA and expanding the knowledge about the different aspects of these conditions. Finally, the results presented in Supplemental Figure 1 demonstrate the similarity of the microbial profiles of amplified and non-amplified samples.

Another limitation of this study is the sample size. Nevertheless, Shin et al.^10^ critically reviewed the 12 peer-reviewed articles that specifically used different NGS technologies to assess the intracanal polymicrobial communities from 2010 to 2017. The total sample size ranged from 7 to 48 samples, with a mean of 19 samples, but only five articles presented a sample size higher than that of this study.

The knowledge raised by this study is the root canal microbiome complexity, the “common denominators” of root canal infections and that symptoms impact the root canal microbiome. Moreover, the virulence properties of identified taxa remain unknown.

### Ethical approval

All procedures performed in studies involving human participants were in accordance with the ethical standards of the institutional and/or national research committee and with the 1964 Helsinki Declaration and its later amendments or comparable ethical standards.

### Informed consent

Informed consent was obtained from all individual participants included in the study.

## Supplementary information

Supplementary file1

## Data Availability

Datasets related to this article can be found at https://homings.forsyth.org/Genus%20probe%20list%20for%20website_v2.0.pdf.
