# Supplementary material for: The apical root canal system microbial communities determined by next-generation sequencing
Source: Sci Rep. 2020 Jul 2;10:10932. doi: 10.1038/s41598-020-67828-3 (PMC7331743; doi:10.1038/s41598-020-67828-3)
Supplement: Supplementary file 1 — Supplementary file1 [file 41598_2020_67828_MOESM1_ESM.pdf]

The apical root canal system microbial communities determined by next-generation sequencing.

Luciana Carla Neves de Brito[a], Janet Doolittle-Hall[b], Chun-Teh Lee [c], Kevin Moss[b], Warley Luciano Fonseca Tavares[d], Antônio Paulino Ribeiro Sobrinho[d], Flávia Rocha Fonseca Teles[e]

[a] University of Itaúuna School of Dentistry, Itaúna, MG, Brazil

[b] Dental Research/Center for Oral Systemic Diseases, University of North Carolina at Chapel Hill School of Dentistry, NC, USA

[c]Department of Periodontics and Dental Hygiene, The University of Texas Health Science Center at Houston School of Dentistry, Houston, TX, USA

[d] Department of Operative Dentistry, School of Dentistry, Federal University of Minas Gerais, Belo Horizonte, MG, Brazil

[e] Department of Periodontology, University of North Carolina at Chapel Hill School of Dentistry, NC, USA

Correspondence to: warleyt@hotmail.com

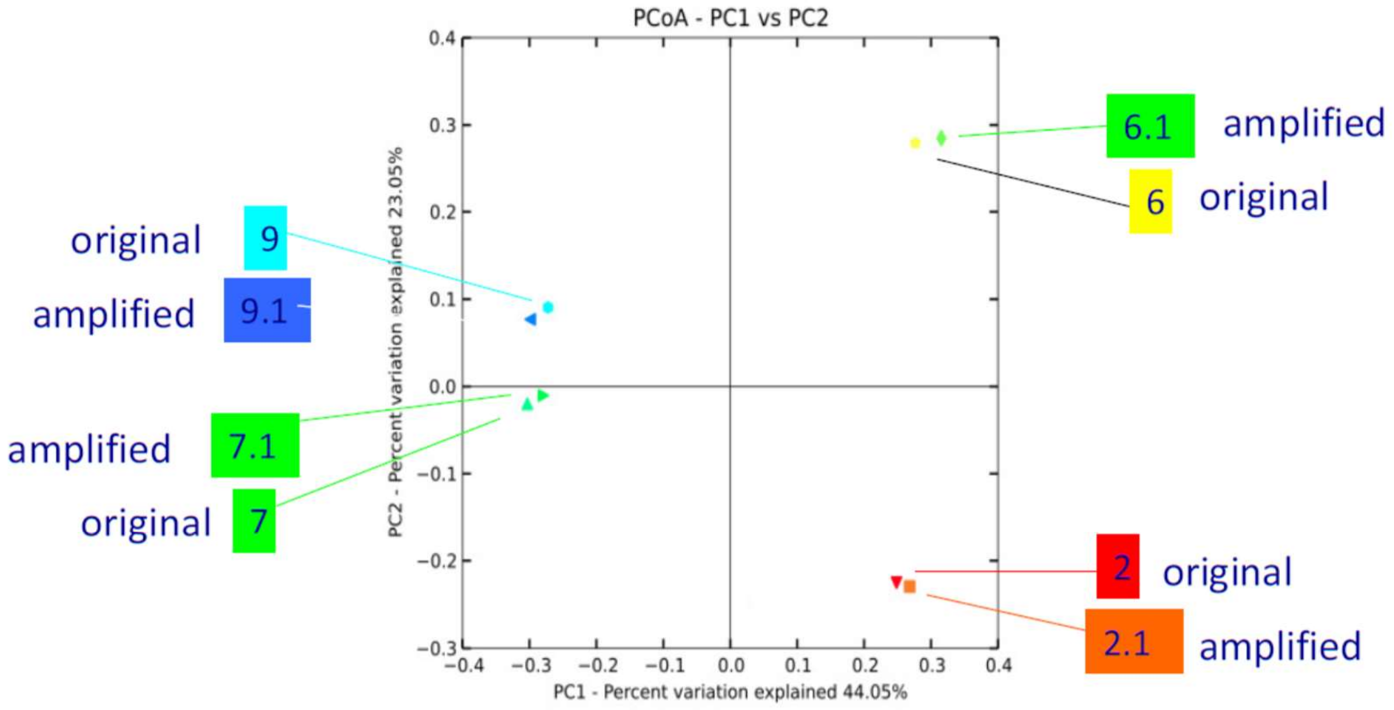

Supplemental Figure 1: Principal coordinate analysis (PCoA) based on unweighted UniFrac distances obtained from the QIIME analytical pipeline comparing four samples that were amplified by MDA (samples number 2.1, 6.1, 7.1, 9.1) to their non-amplified source sample (2, 6, 7, 9, respectively).

Supplemental Fig. 2

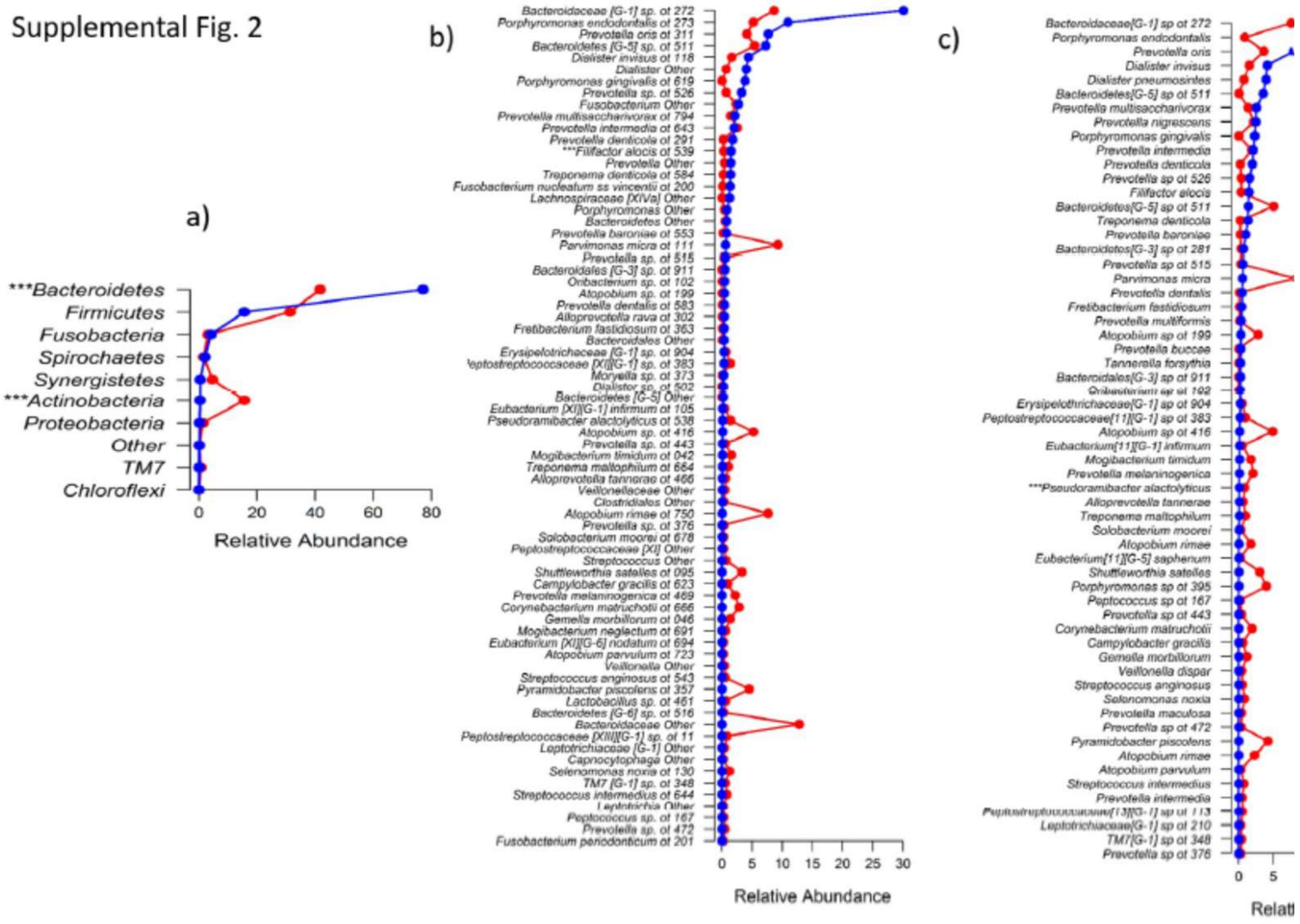

Supplemental Figure 2: Line plots of the mean microbial composition of the samples in which a cavity was open (blue) or closed (red) at the time of sampling, at the phylum (a), genus (b) and species levels (c). Graphs show mean relative abundance for phyla that were 0.01% different, genera that were 0.1% different, and species that were 0.2% different. Taxa were sorted according to relative abundance in the positive group. ***Taxa with statistically significant differences ($p \leq 0.05$).

Supplemental table

Clinical Parameters

Amplified sample	open/closed cavity	presence/ absence lesion	lesion size small/large	symptomatic/ asymptomatic	presence/ absence sinus tract	Male/ female	Age	Single /multiple root
L2	closed	absence	-	asymptomatic	absence	F	32	multi
L6	closed	absence	-	symptomatic	absence	F	26	multi
L7	open	presence	large	symptomatic	absence	M	31	multi
L9	open	presence	small	asymptomatic	absence	F	56	multi
L10	closed	presence	large	asymptomatic	absence	F	39	multi
L16	closed	absence	-	asymptomatic	absence	M	56	multi
L20	open	presence	small	symptomatic	absence	F	53	single
L24	closed	absence	-	asymptomatic	absence	M	69	single
L25	closed	presence	large	symptomatic	presence	M	39	multi
L28	closed	presence	large	symptomatic	absence	F	21	multi
L29	closed	absence	-	asymptomstic	absence	F	14	multi
L34	closed	presence	small	symptomatic	presence	F	41	multi
L36	open	presence	large	symptomatic	absence	F	43	multi
L40	closed	presence	small	symptomatic	absence	M	65	single
L42	closed	presence	large	symptomatic	absence	F	12	multi
